# Experimental study on anti-drift shield curtain device for soybean (*Glycine max*)-maize (*Zeal mays*) strip intercropping weed sprayers

**DOI:** 10.1371/journal.pone.0318683

**Published:** 2025-03-31

**Authors:** Yuxuan Jiao, Qingqing Zhou, Tao Sun, Chen Cai, Longfei Cui, Zhu Sun, Yongkui Jin, Wei Kong, Suming Ding, Xinyu Xue

**Affiliations:** 1 Nanjing Institute of Agricultural Mechanization, Ministry of Agriculture and Rural Affairs, Nanjing, Jiangsu, China; 2 Sino-US Joint Laboratory for Pesticide Application Technology, Nanjing, Jiangsu, China; Canakkale Onsekiz Mart University, TÜRKIYE

## Abstract

Soybean-maize strip intercropping is an important method to increase soybean oil self-sufficiency and promote sustainable agricultural development, which has rapidly developed in China in recent years. However, the herbicides used in the post-emergence weeding stage of soybean and maize can inhibit each other's growth, and herbicide drift can easily lead to crop yield reduction or even death. To reduce the drift risk of soybean-maize strip intercropping sprayers during the post-emergence weed control stage, we explored the efficacy of shield curtain device in reducing drift through wind tunnel and field trials. The study found that when only two sides of shield curtains were used to cover the spray area in the wind tunnel test, the maximum drift rate was 2.18% under the influence of lateral airflow, while drift was more severe under the influence of inter-row airflow with a maximum drift rate of 14.99%. The drift rate far exceeded the threshold causing herbicide damage on soybeans and maize. In the field test, using shield curtains on the front, back, and both sides of the spray area could obtain a maximum spray drift rate of 0.98% and a minimum deposition rate of 57.87%. This approach could reduce the drift rate by more than 88.5% and increase the deposition rate by more than 47.2% compared to not using the shield curtain device. The study can offer valuable reference and data support for the research of anti-drift technology for soybean-maize strip intercropping sprayers and provide ideas for designing shield devices.

## Introduction

China is the world's largest importer of soybeans, with annual soybean imports accounting for an average of about 60% of the world soybean trade. The low self-sufficiency rate of soybeans has become a key issue affecting China’s food security [1,2]. Under the dual background of high demand for soybean expansion and land competition between soybean and maize due to crop growth cycle and economic factors, the soybean-maize strip intercropping model was developed [3,4]. The model intercrops 2-4 rows of maize and 2-6 rows of soybeans in strips. This approach leverages the advantages of maize side rows to increase soybean light exposure and utilizes soybean rhizobacteria to fix nitrogen to reduce the need for fertilizer, ultimately achieving stable maize yields and an additional soybean crop in the same field. This model has become an important strategy to alleviate the competition between soybeans and maize for land in China and reduce the country's reliance on soybean imports [5–7].

Soybean-maize strip intercropping stabilizes maize yields and increases soybean yields, but it presents new challenges for full mechanization. Post-emergence herbicide spraying is a crucial issue that needs urgent resolution. Maize is a monocotyledonous grasses crop, while soybean is a dicotyledonous legume crop. These two crops require different herbicides, which can hinder each other's growth [8–10]. Herbicide drift during spraying can easily lead to crop damage, such as dwarfing, yellowing, and wilting, which poses a significant threat to the safe production of maize and soybean [11–13].

Commonly used methods to reduce drift in plant protection application operations include increasing droplet size, utilizing auxiliary airflow, and setting up shield devices [14,15]. Methods to increase the droplet size involve using anti-drift nozzles, adding anti-drift additives, etc., and the findings from previous research can be applied in soybean-maize strip intercropping [16–19]. Auxiliary airflow is employed to enhance droplet penetration and guide rapid deposition into the target area to reduce drift. Widely used machines based on this method are mainly airflow-assisted sprayers [20–24]. Shield devices are used to isolate the external environment and spraying areas to reduce drift. For soybean-maize strip intercropping anti-drift technology, isolating maize and soybeans is crucial and necessary. Setting up shield devices is the preferred approach to reduce drift which isolates maize and soybeans while also minimizing the impact of external factors on drift. Considering the major demand and cost issues, this study focused on shield devices. Ozkan et al. [25] discovered that wind speed was no longer the primary factor influencing drift after using the shield, and the double circular shield exhibited the best performance with a 59% reduction in drift after comparing nine types of shields. Sidahmed et al. [26] compared the double foil, symmetrical double foil, and symmetrical triple foil shields, finding that using these shields resulted in a 48% or greater reduction in drift compared to not using a shield. The symmetrical triple foil shield obtained the best performance with a 61% reduction in drift. Currently, the shield device structures reported cannot be directly applied in soybean-maize strip intercropping, and there is limited research on anti-drift technology for soybean-maize strip intercropping sprayers. This paper investigates the structure of shield curtain device and its function on drift reduction through wind tunnel and field trials to ensure that the drift rate of herbicides in the actual weeding operation is less than the value that can cause herbicide damage to maize or soybeans [11]. This research can serve as a reference and provide ideas for the development of soybean-maize strip intercropping sprayer drift control technology.

## Materials and methods

### Wind tunnel test

The test was conducted in an NJS-1 type plant protection wind tunnel (non-standard equipment, developed by the Nanjing Mechanization Research Institute of Agriculture, Ministry of Agriculture. The test section is 7.5 m long ×  1.2 m wide ×  1.8 m high, with a wind speed that can be continuously adjusted from 0.5 to 10 m/s. The airflow turbulence is maintained at less than 1%. The wind tunnel is equipped with a complete spray system, including a plunger pump, a water tank, two pressure gauges, a spray rod and other necessary equipment. The test devices mainly used included an anemometer (with a range of 0 ~  50 m/s and an accuracy of ± 2%, Kanomax, Japan), a 722N visible spectrophotometer (INESA Analytical Instrument Co., Ltd., Shanghai, China), Allura Red AC (with a purity of 85%, Beijing Solarbio Science & Technology Co., Ltd., Beijing, China), a one-millionth balance (with a precision of 0.0001 g and a range of 100 g), an electronic scale (with a precision of 0.1 g and a range of 30 kg), paper cards (80 ×  80 mm), syringes, plastic bags, collection racks, disposable gloves, and so on.

**Fig 1:**
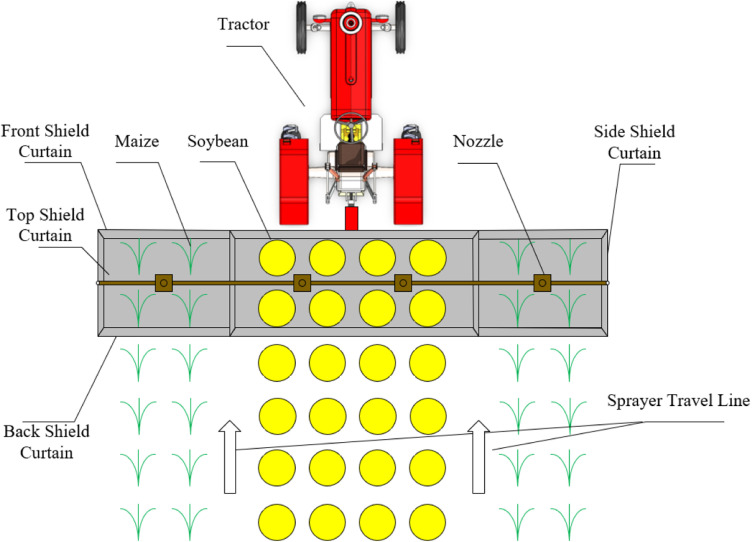
Schematic diagram of shield curtain device of the soybean-maize strip intercropping sprayer.

Take 4 rows of soybeans +  2 rows of maize strip intercropping mode as an example. The design of shield curtain device serves two main purposes. One aim is to separate the soybean rows from the maize rows and the other is to minimize the impact of airflow on herbicide drift. To achieve this, shield curtains were set up on both sides of the two maize rows, effectively separating the two crops and reducing the influence of lateral airflow on drift at the same time. However, only setting up side shield curtains is insufficient. This is because the inter-row airflow, parallel to the sprayer's direction of movement, can carry herbicides to areas without side shield curtains and drifting by external factors. Therefore, it is essential to set up front and back shield curtains, with a height greater than that of the crops to prevent crop damage. Additionally, to prevent small droplets from floating upward out of the shield curtain area and drifting, a top shield curtain must also be installed. In conclusion, the schematic of the shield curtain device setup is shown in Fig 1.

The shield curtain device arrangement in the wind tunnel referred to Fig 1, utilizing PVC transparent soft curtains. The direction parallel to the wind tunnel airflow was considered the front and back direction, while the direction perpendicular to the wind tunnel airflow was the left and right direction. An anti-drift nozzle LANAO FP 90-03 was used, spraying vertically downward and positioned 0.4 m above the wind tunnel floor. The left and right shield curtains were in direct contact with the ground, while the front and back shield curtains needed to be higher than the crop height during the post-emergence weed control period to avoid scratching the crop (approximately 10-40 cm during the 3-5 leaf stage of maize). Therefore, the front and back shield curtains were positioned 0.5 m above the ground. Ultimately the left and right shield curtains were 0.8 m wide and 0.8 m high, the front and back shield curtains were 0.8 m wide and 0.3 m high and the top shield curtain was 0.8 m long and 0.8 m wide. The vertical centerline of the nozzle and the shield curtain device coincided with the vertical centerline of the wind tunnel.

**Fig 2:**
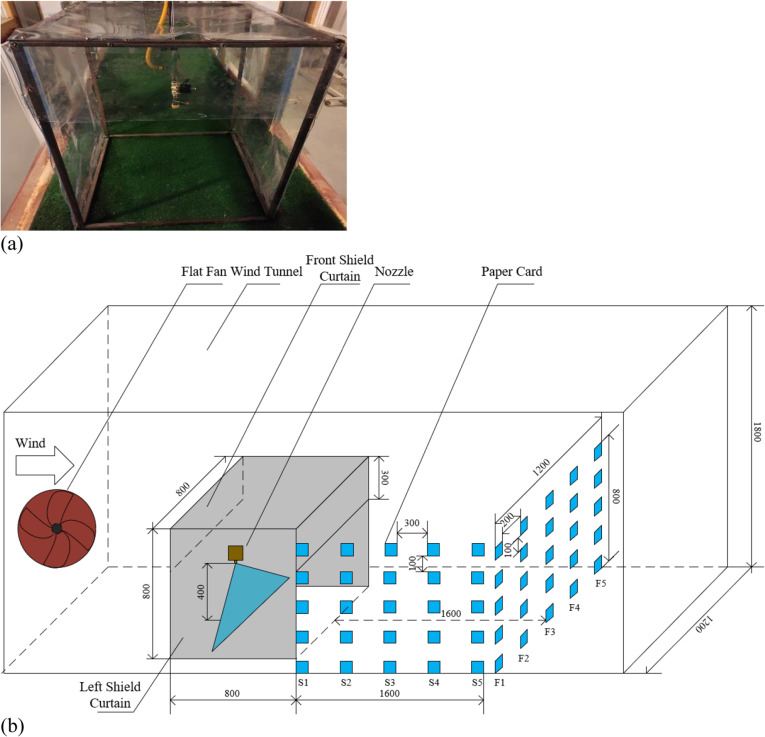
Shield curtain device and sampling arrangement. (a) shield curtain device; (b) sampling arrangement.

Paper cards were used to collect drift droplets. Front sampling was to collect drift droplets 2 m downwind of the nozzle with a sampling height of 0-0.8 m. The vertical sampling interval of the paper cards was 10 cm, and the horizontal interval was 20 cm. A total of five columns of paper cards were arranged with a length of 1.2 m. Facing the wind tunnel, the paper card columns were named as columns F1 ~ F5 from left to right, with paper cards numbered 1 ~ 5 from bottom to top. Column F3 was in a straight line with the nozzle. Side sampling was to collect drift droplets on the left side of the shield curtain device, and paper cards were arranged on the wall of the wind tunnel at a sampling height of 0-0.8 m. The vertical sampling interval of the paper cards was 10 cm, and the horizontal interval was 30 cm. A total of five columns of paper cards were arranged with a length of 1.6 m from the shield curtain device. The paper card columns were named as columns S1 ~ S5 from near to far from the shield curtain device, and the outside of paper cards in column S5 were in a straight line with the front sampling paper cards. The spray solution used was Allura Red AC with a concentration of 1 g/L, and a solenoid valve was used to ensure that the spraying time was fixed at 10 seconds for each test. The arrangement of the shield curtain device and the sampling are shown in Fig 2.

**Fig 3:**
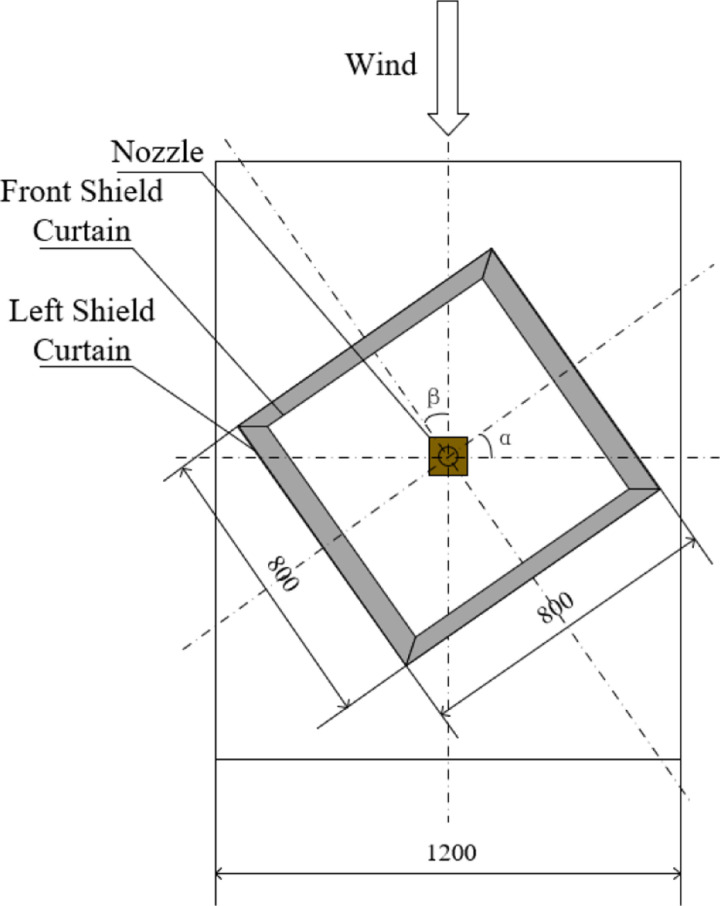
Shield curtain device deflection angle.

The occasional gust wind speed may exceed 3 m/s, which is the permissible operating wind speed of the sprayer during field operations [27]. In this study, the fixed spray pressure was 0.3 MPa, and the wind tunnel wind speed was 5 m/s. To evaluate the anti-drift performance of the shield curtain device against inter-row airflow and lateral airflow, drift droplets will be collected at wind direction angles of 0° and 90°. The wind tunnel airflow was simulated as inter-row airflow in the direction of the sprayer's travel at a 0° wind direction angle and as lateral airflow at a 90° wind direction angle. Since the wind direction of the wind tunnel cannot be altered, the corresponding wind direction angle β can be obtained by adjusting the deflection angle α of the shield curtain device. The deflection angle of the shield curtain device is shown in Fig 3, where the wind direction angle is 90° when the shield curtain device is deflected by 90°. The single-group test was conducted three times, and the results were averaged. Finally, the nozzle type and spray parameters are set as shown in Table 1, and the spray performance parameters of the nozzle are shown in Table 2.

**Table 1:** Spray parameters.

Nozzle Type	Pressure/MPa	Wind speed/m·s^−1^	Wind direction angle/°
LANAO FP 90-03	0.3	5	0°,90

**Table 2:** LANAO FP 90-03 spray performance parameters.

Nozzle Type	Flow Rate/L·min^−1^	DV_10_/μm	DV_50/_μm	DV_90_/μm
LANAO FP 90-03	1.2 ± 0.02	272.18 ± 2.60	534.95 ± 1.29	826.84 ± 5.12

Note: The data represent mean ±  standard deviation and is the same as bellow. The method of obtaining the nozzle spray performance parameters was described in the part 2.2 Atomization Characteristic Test of the paper [28].

### Field trials

**Fig 4:**
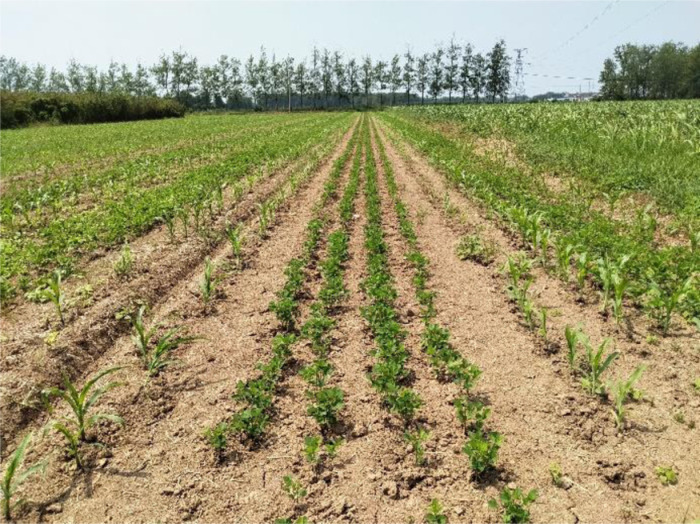
Soybean-maize strip intercropping field.

Test object: Soybean and maize in the standard “4+2” soybean-maize strip intercropping fields. The soybean variety was Su Huang No. 1 (Jiangsu Academy of Agricultural Sciences, Jiangsu, China), and the maize variety was Su Yu 39 (Biocentury Transgene (China) Co. Ltd., Guangdong, China). Animal manure such as buffalo dung and chicken manure was well rotted and applied as the soil base fertilizer when sowing seeds. The main components of these organic fertilizers are nitrogen, phosphorus, potassium and other trace elements. Maize rows were spaced 40 cm apart, soybean rows were spaced 30 cm apart, and the distance between the two crops were 70 cm, as shown in Fig 4.

Herbicides: The soybean herbicide was 10% quizalofop-p-ethyl emulsifiable concentrate (Shandong Agricultural Biotechnology Co., Ltd., Shandong, China) with a dosage of 40 mL/667m^2^. The maize herbicide was 20% fluroxypyr-meptyl emulsifiable concentrate (Anhui Huaxing Chemical Industry Co., Ltd., Anhui, China) with a dosage of 30 mL/667m^2^. Both herbicides were mixed with Allura Red AC at a concentration of 1 g/L respectively.

Main materials and instruments: polyester disk (φ90 mm), portable anemometer Kestrel 5500, one ten-thousandth balance, sampling rod, disposable gloves, etc.

**Fig 5:**
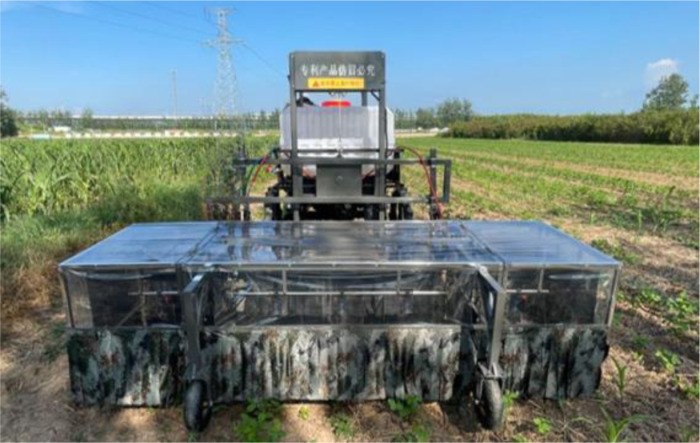
3WPZ-600 soybean-maize strip intercropping special sprayer.

Test equipment: 3WPZ-600 soybean-maize strip intercropping special sprayer (Sangpu Agricultural Machinery (Changzhou) Co., Ltd., Jiangsu, China), as shown in Fig 5. Combined with the soybean and maize row spacing in standard “4 + 2” soybean-maize strip intercropping fields, the design of the shield curtain device was similar to that of the wind tunnel test. The sprayer was equipped with five anti-drift nozzles LANAO FP 90-03 placed at the midpoint of the rows. The middle soybean rows were equipped with 3 nozzles and maize rows on both sides were equipped with 2 nozzles. To further reduce drift, cotton strips were added to the front and back shield curtains at 0.5 m above the ground up to the floor.

Test date: August 19, 2023.

Meteorological conditions: wind speed 0 ~  4.7 m/s, average wind speed 1.7 m/s, main wind direction angles 0° ~  16° and 83° ~  102°; air temperature 29.2 ~  35.6 °C, average temperature 31.6 °C; humidity 63 ~  80.6%, average humidity 71.2%.

Test site: Li Ji Township, Guannan County, Lianyungang City, Jiangsu Province, China (Latitude 34.054° N, longitude 119.268° E).

**Fig 6:**
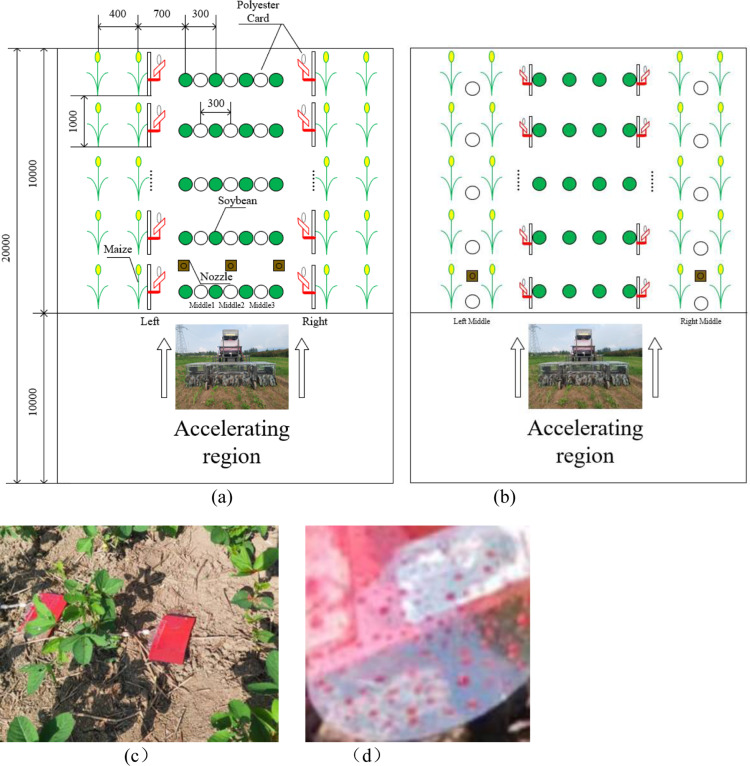
Sampling schematic. (a) Soybean herbicide spraying; (b) Maize herbicide spraying; (c) Samples of soybean deposition; (d) Polyester disk with deposition.

The amount of liquid applied was 30 L/667m². The traveling speed was 1 m/s, and the spray pressure was 0.3 MPa. The single traveling distance was set to 20 m. The first 10 m was the machine acceleration zone, and the second 10 m was sampled with a sampling interval of 1 m for a total of 10 sampling points. When spraying the soybean herbicide, only open nozzles above soybean rows and arrange polyester disks to collect deposited droplets at the midpoint of adjacent soybeans on the ground with polyester disks placed horizontally upward, and arrange polyester disks to collect drift droplets next to the outside of maize adjacent to soybean rows with polyester disks placed vertically facing the soybeans and the sampling height equal to that of the maize canopy. Similarly, when spraying the maize herbicide, only open nozzles above maize rows and arrange polyester disks in the midpoint of the maize on the ground and on the outside of soybeans adjacent to the maize row, sampling height equal to that of soybean canopy. The samples on the left side of the forward direction of the sprayer were named Left 1 ~ Left 10, and the samples on the right side of the forward direction were named Right 1 ~ Right 10; Soybean deposition samples were named middle 1 to middle 3 from left to right, and maize deposition samples were middle left and middle right. The sampling arrangement is as shown in Fig 6. The shield curtain device was removable, and tests were conducted under the conditions with and without the shield curtain device.

### Sample processing and calculations

After the spraying process, gloves should be worn to prevent contamination when retrieving the paper cards or polyester disks from the wind tunnel or field. Once the droplets on these samples have dried, each paper card or polyester disk should be placed into a separate plastic bag. The location of each sample must be carefully recorded on the corresponding plastic bag label. The samples should then be transported to the laboratory for further analysis and processing.

The paper cards were scanned and analyzed using DepositScan software to quantify droplet deposition [29]. To elute the polyester disks, 12 mL of distilled water was injected into each plastic bag containing a disk, followed by mechanical shaking to facilitate elution. The absorbance of the eluent was measured using a visible spectrophotometer, and the concentration of Allura Red AC was determined by referencing a pre-established “concentration-absorbance” standard curve for this dye. The deposition rate of spray drift per unit area of the polyester disk was then calculated using formula (1), and the spray drift efficiency was determined according to formula (2).


$$\text{SD}=\frac{(C_{S}-C_{B})\times \text{F}\times V}{C_{M}\times A}$$ (1)

where:

SD - spray drift/deposition amount per unit area of polyester disk, mL/cm^2^;

C_S_ - Concentration of sample eluent, mg/L;

C_B_ - Blank sample eluate concentration, mg/L;

F - calibration factor, 1 for polyester disks;

V - Volume of distilled water used to elute the sample, mL;

C_M_ - Spray stock solution concentration, mg/L;

A - Area of polyester disk, cm^2^.


$$\text{SDR}=\frac{SDV\times 100\%}{V_{S}}$$ (2)

where:

SDR - Spray Drift Rate, %;

SDV - Total spray drift volume, mL;

V_S_ - Spray application volume, mL.

## Results and analysis

### Wind tunnel test

**Fig 7:**
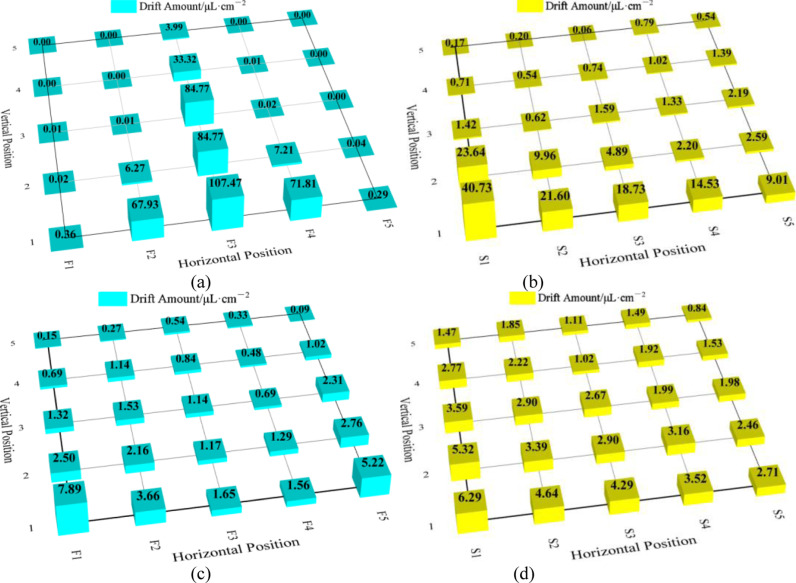
Front and side sampling deposits at 0° and 90° guard curtain deflection angles. (a) front deposition at 0° guard curtain deflection angle; (b) Side deposition at 0° guard curtain deflection angle; (c) Front deposition at 90° guard curtain deflection angle; (d) Side deposition at 90° guard curtain deflection angle.

The front and side drift amount at the 0° and 90° wind direction angles are shown in Fig 7 and the drift rates are shown in Table 3.

**Table 3:** Drift rate of droplets at 0° and 90° guard curtain deflection angles/%.

Positions and angles	Front drift rate at 0° guard curtain deflection angle	Side drift rate at 0° guard curtain deflection angle	Front drift rate at 90° guard curtain deflection angle	Side drift rate at 90° guard curtain deflection angle
**Drift Rate**	14.99	5.16	1.36	2.18

At the 0° wind direction angle, the wind tunnel airflow simulated the airflow between crop rows. Front-drifting droplets deposited more as they got closer to the ground vertically and increased from the sides to the center horizontally with a drift rate of 14.99%. 91.88% of the droplets deposited within the 0.8 m width of the shield curtain device. Because the spray nozzle droplet distribution shows the structure that more droplets deposit in the middle and fewer on both sides, and the anti-drift nozzle produces droplets with coarse even ultra-coarse droplet sizes in general. Most of the droplets deposited rapidly downward under the impact of pressure upon leaving the nozzle. Although droplets with medium sizes will move along the airflow direction, they also gradually deposited downwards. Only a very small number of fine droplets will float upwards and deposit. Side-drifting droplets deposited more as they got closer to the ground vertically and to the shield curtain device horizontally with a drift rate of 5.16%. Because airflow will bounce back or spread out to the sides when hitting the shield curtain device. Some of the droplets were carried by the airflow to the walls of the wind tunnel and deposited mainly in the area close to the shield curtain device. It can be observed that the droplets drifted more under the 0 ° wind direction angle, especially between the rows. Fig 7 shows that there was little droplet deposition within the shielding height of the front and back curtains, but the gap of 50 cm from the ground gave droplets ample space to drift. Once these droplets leave the shield curtain area, they can be easily transported to neighboring crops by lateral airflow and cause damage. Therefore, the front and back shield curtains need to provide as much shelter as possible without damaging the crop.

At the 90° wind direction angle, the wind tunnel airflow simulated the lateral airflow. Front-drifting droplets deposited more as they got closer to the ground vertically and decreased from the sides to the center horizontally with a drift rate of 1.36%. Because droplets could not deposit through the curtains within the 0.8 m width of the shield curtain device, and deposited with the airflow on both sides beyond the width of the shield curtain device. The results for side-drifting droplets were similar to those at the 0° wind angle with a drift rate of 2.18%. Few droplets drifted at the 90° wind direction angle. Most of the droplets deposited in the shield curtain area, but some airflow bouncing off the shield curtains or wind tunnel wall entered through the gap between the front and back curtains and the ground, causing droplet drift. At the same time, there may be a very small number of droplets splashing out of the shield curtain area and being carried by the airflow to drift.

### Field trials

The drift and deposition results of soybean and maize are shown in Table 4.

**Table 4:** Drift and Deposition Results of Soybean and Maize.

Maize			Soybean		
Drift Amount/μL·cm^−2^					
Sampling Point	Without Curtains	With Curtains	Sampling Point	Without Curtains	With Curtains
1	75.04 ± 1.11	9.06 ± 0.99	1	49.21 ± 1.58	5.07 ± 0.15
2	86.06 ± 6.17	8.25 ± 0.66	2	54.49 ± 4.56	5.22 ± 0.33
3	67.10 ± 5.90	8.54 ± 0.18	3	44.85 ± 3.87	5.23 ± 0.31
4	74.33 ± 4.87	8.76 ± 0.28	4	44.08 ± 3.42	5.30 ± 0.83
5	111.49 ± 11.84	9.61 ± 0.35	5	46.55 ± 3.96	5.66 ± 0.56
6	66.75 ± 4.20	11.62 ± 0.22	6	42.69 ± 1.56	5.90 ± 0.10
7	102.98 ± 9.72	11.85 ± 0.55	7	38.22 ± 4.21	5.29 ± 0.13
8	110.41 ± 2.98	7.75 ± 0.87	8	49.47 ± 0.52	5.02 ± 0.57
9	92.22 ± 1.84	9.46 ± 1.07	9	41.27 ± 0.19	4.43 ± 0.23
10	88.76 ± 5.14	7.92 ± 0.59	10	41.12 ± 2.94	5.30 ± 0.26
**Average**	87.51 ± 15.94	9.28 ± 1.36	**Average**	45.19 ± 4.59	5.24 ± 0.37
**Coefficient of Variation**	18.22	14.60	**Coefficient of Variation**	10.16	7.03
**Drift Rate/%**	9.28	0.98	**Drift Rate/%**	7.19	0.83
**Deposition Amount/μL·cm** ^**−2**^					
**Sampling Point**	**Without Curtains**	**With Curtains**	**Sampling Point**	**Without Curtains**	**With Curtains**
1	113.70 ± 9.49	203.65 ± 10.30	1	119.74 ± 9.95	177.00 ± 7.57
2	115.00 ± 8.80	173.22 ± 7.12	2	126.42 ± 7.58	171.35 ± 6.52
3	118.38 ± 13.85	181.21 ± 7.03	3	152.15 ± 11.52	206.76 ± 6.41
4	131.54 ± 7.30	207.53 ± 3.13	4	103.98 ± 9.88	186.28 ± 6.14
5	132.67 ± 13.84	181.85 ± 11.88	5	118.09 ± 9.55	143.62 ± 4.55
6	131.11 ± 5.58	186.38 ± 10.28	6	96.97 ± 7.88	212.65 ± 12.84
7	108.58 ± 7.69	222.25 ± 3.24	7	148.73 ± 13.59	177.06 ± 12.06
8	90.77 ± 1.15	172.95 ± 6.93	8	150.15 ± 4.19	172.87 ± 9.64
9	133.22 ± 11.39	194.64 ± 5.90	9	111.23 ± 10.14	191.02 ± 2.27
10	97.47 ± 8.13	193.30 ± 5.37	10	108.98 ± 10.55	181.16 ± 6.58
**Average**	117.24 ± 14.41	191.70 ± 15.06	**Average**	123.65 ± 19.14	181.98 ± 18.36
**Coefficient of Variation**	12.29	7.85	**Coefficient of Variation**	15.48	10.09
**Deposition Rate/%**	37.28	60.96	**Deposition Rate/%**	39.32	57.87

Note: Soybean and maize drift were averaged from the left and right sides of each sampling point; soybean deposition was averaged from the middle 1, middle 2, and middle 3 of each sampling point; and maize deposition was averaged from the left middle and right middle.

The average drift amount of 10 sampling points of maize was 87.51 μL/cm^2^ and the drift rate was 9.28% without the use of shield curtain device; the average drift amount was 9.28 μL/cm^2^ and the drift rate was 0.98% with the use of shield curtain device. The drift amount was reduced by 78.23 μL/cm^2^, and the drift rate was reduced by 89.4%. The average drift amount of 10 sampling points of soybeans was 45.19 μL/cm^2^ and the drift rate was 7.19% without the use of shield curtain device; the average drift amount was 5.24 μL/cm^2^, and the drift rate was 0.83% with the use of shield curtain device. The drift amount was reduced by 39.95 μL/cm^2^ and the drift rate was reduced by 88.5%.

The average deposition amount of 10 sampling points of maize was 117.24 μL/cm^2^ and the deposition rate was 37.28% without the use of shield curtain device; the average deposition amount was 191.7 μL/cm^2^ and the deposition rate was 60.96% with the use of shield curtain device. The deposition amount increased by 74.46 μL/cm^2^ and the deposition rate increased by 63.5%. The average deposition amount of 10 sampling points of soybeans was 123.65 μL/cm^2^ and the deposition rate was 39.32% without the use of shield curtain device; the average deposition amount was 181.98 μL/cm^2^ and the deposition rate was 57.87% with the use of shield curtain device. The deposition amount was increased by 58.33 μL/cm^2^ and the deposition rate was increased by 47.2%.

Based on the above results, Airflow was identified as the dominant factor influencing spray drift. The implementation of the shield curtain device markedly reduced the impact of external airflow on spray distribution, particularly within inter-row and lateral airflow regions. A reduction in the coefficient of variation across sampling points indicated enhanced stability of drift and deposition, minimizing environmental interference. Wind tunnel experiments further demonstrated that integrating cotton strips into the front and back shield curtains significantly enhanced drift reduction, validating the efficacy of this modification. These findings provide critical experimental evidence for optimizing anti-drift technologies in soybean–maize strip intercropping sprayer systems.

## Discussion

The latest research reported that Wang et al. [30] used hoods in soybean rows to reduce drift, and they discovered that the height of the hood above the ground was positively correlated with drift. A height difference of 10 cm caused about 30 more droplets per square centimeter drifting. The designed spray system achieved a 40% to 50% soybean deposition rate and a 50% to 68% maize deposition rate. However, the study had several limitations. First, soybean plants were not positioned outside the maize rows during the field trial, and the analysis was confined to drift from the maize rows toward the soybeans within the inter-row area. In actual field conditions, maize is flanked by soybeans on both sides, and drift occurs not only toward the inner soybeans but also toward those on the outer edges. Second, an axial fan was employed to generate lateral airflow without altering the wind direction angle, but the wind directions during actual field operations are inherently dynamic and variable. These changes in airflow direction can significantly influence droplet movement, leading to notable variations in spray drift and deposition [31,32]. The other latest research reported that Wang et al. [33] designed a split-belt boom sprayer for soybean-maize strip intercropping. Shelter cloth was placed between maize and soybeans and on the sides and back of the spray boom. The anti-drift rate was 92.52% compared to not using shelter cloth. The design concept of the shield devices in the study was similar to our research, and it also showed that a good anti-drift effect can be obtained without the use of a shelter cloth on the front side of the spray boom. The shield curtain device tested in this paper have a good effect on increasing the deposition of crop rows and reducing the drift of non-target areas. The maximum drift rate after using the shield curtain was 0.98%, which was less than the critical value that caused herbicide damage. The minimum critical value for drift deposition rate leading to herbicide damage in maize and soybean were respectively 1.23% and 1.01%, depending on the spray dose [11,34]. Furthermore, one aspect that needs to be considered in subsequent related research is the droplet deposition on the inner wall of shield devices. The droplet deposition amount on the inner wall of shield devices will gradually accumulate to the maximum absorption saturation of the shield devices. These droplets will trickle down to the ground and could lead to the deposition of herbicide residue. Under standard operating conditions, where the spray width of the nozzle remains within the designed range and is unaffected by external factors such as wind speed, direction, or nozzle settings, the spray does not make contact with the inner surface of the shield curtain devices. This is crucial to prevent excessive droplet deposition on the shield curtain devices, which could result in liquid wastage and the potential for other associated risks. However, droplets may come into contact with the shield curtain's inner wall if they are carried by the airflow within the shield or if they first strike the crop and subsequently rebound toward the shield. In other words, optimizing the structure and material of shield devices may become the next step of the work plan. Because we not only ensure the anti-drift effect of shield devices, but also have to consider the lightness and simplicity of the structure, sustainable development and so on.

## Conclusions

Herbicide drift during post-emergence weeding is one of the challenges in soybean-maize strip intercropping. This paper conducted wind tunnel exploratory tests and field validation experiments for the sprayer shield curtain device. The wind tunnel tests revealed that when only the two sides of shield curtains were used, the maximum drift rate under the influence of lateral airflow was 2.18% and the maximum drift rate under the influence of inter-row airflow could reach 14.99% which is far beyond the critical value of causing herbicide damage. It is recommended that the front and back sides of the nozzle need to use shield devices. The final design of shield curtain device was based on the wind tunnel study and front and back curtains were added with the cotton cloth strips up to the ground. It can play a better role in blocking the inter-row airflow without scratching the crop. This approach could reduce the drift rate by more than 88.5% and increase the deposition rate by more than 47.2% compared to not using shield curtain device. The maximum herbicide drift rate after using the shield curtain was 0.98%, which would not cause herbicide damage to the crop. This study can provide ideas and references for the research and solutions for anti-drift technology for strip intercropping sprayer for soybean-maize, as well as many other crops, which could be preferably grown as intercrops.

## Supporting information

S1 Data Data support files. (XLSX)
